# Electrospray ionization–tandem mass spectrometric study of fused nitrogen‐containing ring systems

**DOI:** 10.1002/jms.4870

**Published:** 2022-06-09

**Authors:** Gábor Krajsovszky, Borbála Dalmadiné Kiss, Krisztina Ludányi, István M. Mándity, Dóra Bogdán

**Affiliations:** ^1^ Department of Organic Chemistry Semmelweis University Budapest Hungary; ^2^ Department of Pharmaceutics Semmelweis University Budapest Hungary; ^3^ Artificial Transporters Research Group, Research Centre for Natural Sciences Institute of Materials and Environmental Chemistry Budapest Hungary

**Keywords:** fragmentation, fused *N*‐heterocycles, pyridazino‐indoles, pyridazino‐quinolines, pyrimido‐cinnolines, pyrimido‐quinolines, tandem mass spectrometry

## Abstract

Four fused nitrogen‐containing ring systems were investigated by electrospray ionization–tandem mass spectrometry: Pyridazino‐indoles, pyridazino‐quinolines, a pyrimido‐quinoline derivative and pyrimido‐cinnolines. Fragmentation patterns of these compounds are discussed and compared. Several characteristic cross‐ring fragments were formed mainly on the pyridazine and pyrimidine rings of the ring systems. The connected Cl, NO_2_, Me, Ph and more extended heterocyclic substituents influenced the fragmentation.

## 1 INTRODUCTION

*N*‐containing heterocyclic compounds possess diverse biological activities and frequently present in molecules of interest in medicinal chemistry.^1^, ^2^, ^3^ Among them, the investigated ring‐systems show also several important activities: Pyridazino‐indole heterocycle is a structural element in antihypertensive, platelet‐aggregation inhibitor compounds or benzodiazepine‐receptor ligands.^4^, ^5^, ^6^, ^7^ Pyridazino‐quinolines have topoisomerase‐inhibitor and cytotoxic activites.^8^ Pyrimido‐quinolines are representatives of antioxidant and antimicrobial compounds.^9^, ^10^ Pyrimido‐cinnolines are potent candidates for antimicrobial, anti‐inflammatory and antiplatelet application.^11^, ^12^

The studied compounds were synthesized by Suzuki—condensation ring closure tandem reactions as we described in our previous studies.^13^, ^14^, ^15^, ^16^ In continuation of our work on the mass fragmentation mechanisms of the thieno[3′,2′:4,5]pyrido[2,3‐d]pyridazine ring system,^17^ a detailed study on fused *N*‐heterocycles was performed to understand the fragmentation behaviour of these compounds. This paper reports a study on the fragmentation mechanisms under ESI/MS conditions of pyridazino‐indoles (**1**–**6**), pyridazino‐quinolines (**7**–**12**), a pyrimido‐quinoline derivative (**13**) and pyrimido‐cinnolines (**14**–**15**). The [M + H]^+^ values of the compounds are summarized in Table 1.

**TABLE 1:** [M + H]^+^ values of **1–15**

Compound	[M + H]^+^
**1**	200
**2**	262
**3**	245
**4**	279
**5**	229
**6**	263
**7**	303
**8**	337
**9**	365
**10**	411
**11**	479
**12**	535
**13**	333
**14**	243
**15**	277

## 2 RESULTS AND DISCUSSION

### 2.1 Pyridazino‐indoles

For **1**, three peaks were detected in the MS/MS spectrum: *m*/*z* = 116, 143 and 169. The loss of pyridazine N and CH_3_ (as a methylamine) resulted in the fragment ion at *m*/*z* = 169. Two further cross‐ring fragments by the cross‐ring‐cleavage of the pyridazine ring were identified at *m*/*z* = 116 and 143. The fragmentation pattern of **2** showed the same *m*/*z* = 143 and *m*/*z* = 169 (*m*/*z* = 169: due to the loss of an aniline) fragments as in **1**. An additional fragment at *m*/*z* = 104 is appeared due to fragmentation on the pyridazino‐indole ring system. *m*/*z* = 234 is detected as a loss of CO.

In case of **3** and **4**, the chlorine on the C ring did not influence the fragmentation behaviour. A loss of the nitro group resulted in a fragment ion of *m*/*z* = 199/233. Cross‐ring fragmentation on the pyridazino ring led to *m*/*z* = 144 in **3** and *m*/*z* = 178 in **4**. Similarly to **1**, the loss of methylamine could be detected in the case of these compounds (*m*/*z* = 169 in **3** and *m*/*z* = 203 in **4**).

**FIGURE 1:**
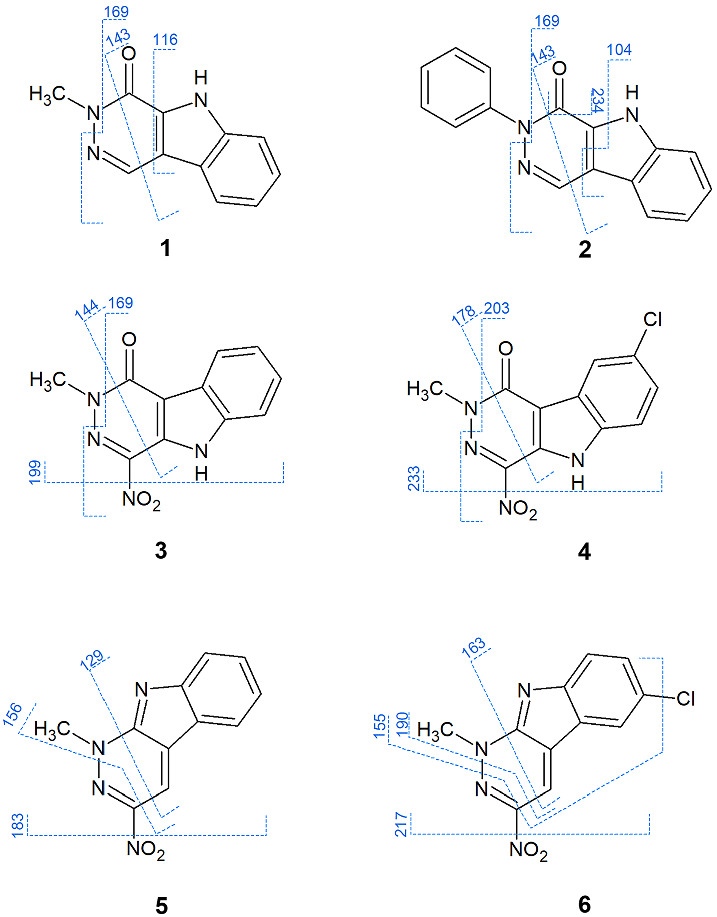
Fragmentation schemes of **1**–**6**

Comparing **5** and **6**, a more pronounced fragmentation occurred in the chlorine‐derivative **6**. The loss of the nitro group was seen in both cases as in **3** and **4** (*m*/*z* = 183 in **5** and 217 in **6**). Cross‐ring cleavages on the pyridazine ring generated the ions at *m*/*z* = 129 and 156 in **5**, and *m*/*z* = 163 and 190 in **6**. The formation of *m*/*z* = 190 was followed by a loss of the chlorine (*m*/*z* = 155) (Figure 1).

### 2.2 Pyridazino‐quinolines

Compound **7** showed a complex fragmentation with several cross‐ring fragments and rupture between the quinoline and the phenylamino substituent (ion of aniline at *m*/*z* = 93 and after the loss of phenyl group from the [M + H]^+^, the remaining cation at *m*/*z* = 226). In **8**, the chlorine‐derivative of **7**, similar fragmentation was observed. Cleavage of the phenylamino/phenyl moiety was also detectable: *m*/*z* = 93 and 244. In both compounds, the methylamine loss was detectable at *m*/*z* = 272 in **7** and *m*/*z* = 308 in **8**. In the N‐2‐phenyl derivative (**9**), different fragmentation occurred in the pyridazino‐quinoline ring system, giving the fragment ions at *m*/*z* = 320 and 244. Between the phenylamino substituent and quinoline ring, the same rupture was seen as in **8** (*m*/*z* = 272). *m*/*z* = 217 could be explained as a formation of an aryne‐type cation from the quinoline. Characteristic *m*/*z* = 77 appeared from the cleavage of the phenyl moiety.

**FIGURE 2:**
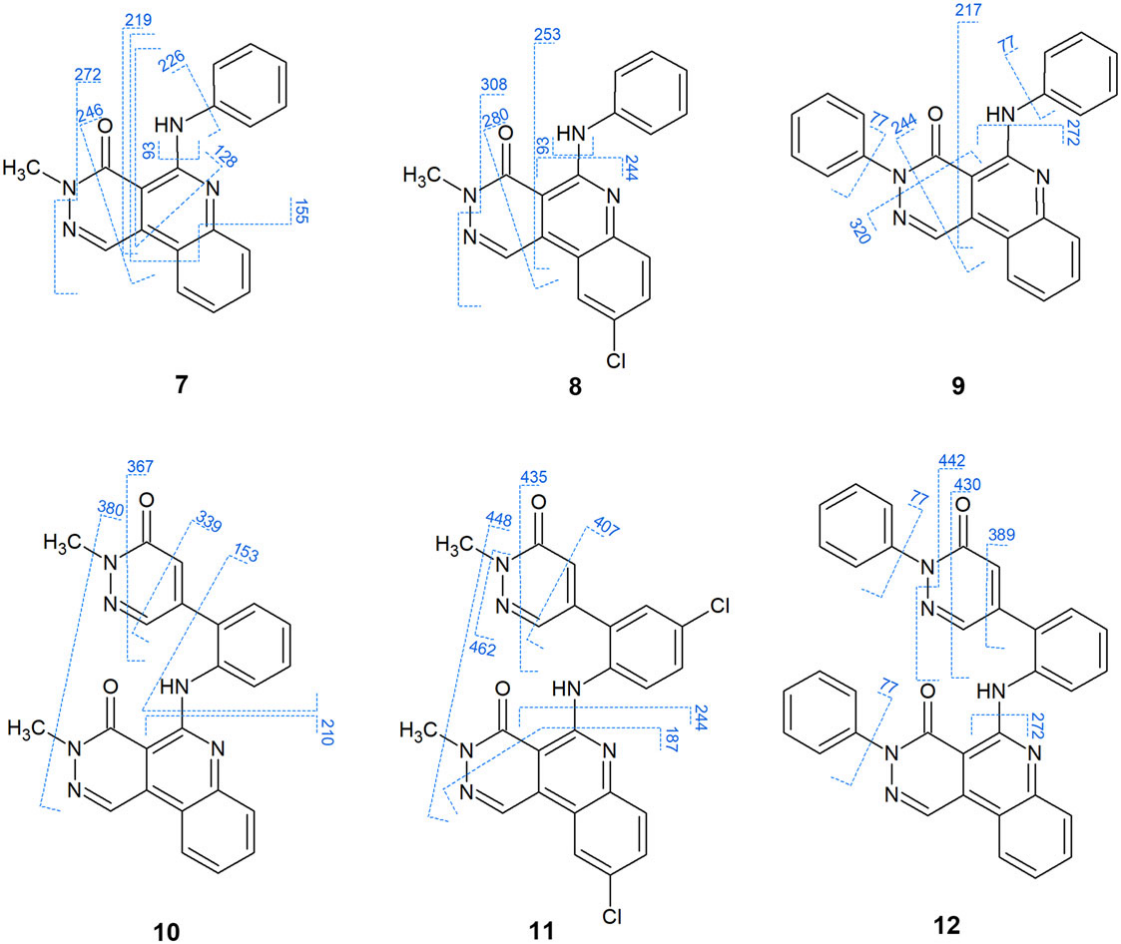
Fragmentation schemes of **7**–**12**

We compared compounds **7–9** with their analogue structures with an extended substituent on the quinoline ring. Rather different fragmentation patterns were observed for **10**–**12**. These three compounds tended to produce fragment ions originated from the rupture on the pyridazine ring connected to the phenylamino moiety. The loss of methylhydrazine and phenylhydrazine resulted in the fragment ions at *m*/*z* = 367 (**10**), 435 (**11**) and 430 (**12**). In case of **10** and **12**, no fragmentation occurred on the pyridazino‐quinoline ring system. The characteristic *m*/*z* = 77 appeared by the cleavage of the phenyl from pyridazino‐quinoline or pyridazine N‐2 in **12**. In **10** and **11**, two methyl groups with the formation of methane provides to peaks *m*/*z* = 380 (**10**) and 448 (**11**) (Figure 2).

### 2.3 Pyrimido‐quinoline

**FIGURE 3:**
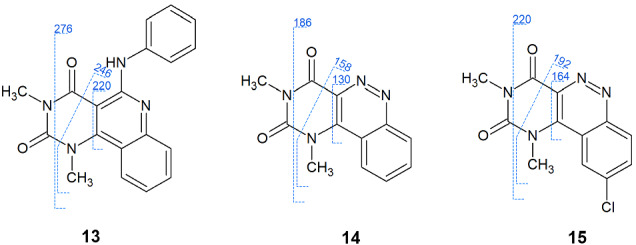
Fragmentation schemes of **13**–**15**

**13** showed fragmentation only on the pyrimidine ring during the MS experiments. *m*/*z* = 276 fragment was detected after the ejection of a H_3_CNC=O. The formyl loss from this fragment was possible; resulted in *m*/*z* = 246. The quinoline ring system could remain in the fragmentation process (*m*/*z* = 220) (Figure 3).

### 2.4 Pyrimido‐cinnolines

**14** and **15** could also fragment via the same bond cleavages as **13** on the pyrimidine ring to produce the detected ions. The cinnoline part appeared at *m*/*z* = 130 (**14**) and 164 (**15**). The cross‐ring fragment ions were *m*/*z* = 186/220; and after formyl loss 158/192 in **14**/**15** (Figure 3).

## 3 EXPERIMENTAL

### 3.1 General

Chemicals were obtained from VWR chemicals (France). All solvents were HPLC grade. All compounds were synthesized at the Department of Organic Chemistry, Semmelweis University, which were published previously.^13^, ^14^, ^15^, ^16^

### 3.2 MS and MS/MS measurements

Agilent 6460 QQQ mass spectrometer with Jet Stream electrospray ion source (ESI/MS) (Waldbronn, Germany) was used to perform MS and MS/MS measurements in positive mode. Samples were dissolved in acetonitrile: Water containing 0.5% formic acid = 1:1, 5 μl samples were injected, flow rate was 0.5 ml/min. Eluent contained acetonitrile: water containing 0.5% formic acid = 1:1.

Capillary voltage was 3500 V, the capillary temperature was kept at 300°C. Quadrupole scanned over the range m/z 50–1000 in MS measurements. In MS/MS measurements product ion scan mode was applied, protonated molecular ions (MH+) were studied. Nebulizer, sheath and collision gas was nitrogen. The set parameters were as follows: Fragment or voltage 135 V, gas flow rate 12 L/min, nebulizer gas flow 45 psi, sheath gas flow 11 L/min, sheath gas temperature (heater) 400°C. The set collision energies are summarized in Table 2. Spectra were evaluated with Agilent MassHunter B 02.01. software.

**TABLE 2:** Collision energies used in tandem mass spectrometry experiments

Compound investigated	Collision energy (eV)
**1**	20
**2**	20
**3**	15
**4**	15
**5**	20
**6**	20
**7**	50
**8**	40
**9**	40
**10**	30
**11**	30
**12**	30
**13**	30
**14**	25
**15**	20

### 3.3 Conclusions

Fifteen nitrogen‐containing ring systems were studied by tandem mass spectrometry. In case of the six pyridazino‐indoles, all compounds showed cross‐ring fragmentation on the pyridazine ring. For the nitro derivatives, as **3**–**6**, the loss of NO_2_ was also seen. In the chlorine‐containing **4** no additional fragments appeared compared to its analogue structure **3**. Contrary to this, the fragmentation of the chlorine‐derivative **6** resulted in a new fragment affected by the chlorine‐substituent. Intensive fragmentation on the whole heterocylic ringsystem was detected for pyridazino‐quinolines **7**–**9**. The other set of pyridazino‐quinolines **10**–**12** with an extended substituent showed fragmentation mainly on the pyridazino substituent connected to the phenylamino moiety. The chlorine‐substituent in **8** and **11** had no effect on the fragmentation. Pyrimido‐quionoline (**13**) and pyrimido‐cinnolines (**14** and **15**) tended to give fragments originated predominantly from the pyrimidine ring.

## Supporting information

Figure S1: CID mass spectrum of **1**
Figure S2: CID mass spectrum of **2**
Figure S3: CID mass spectrum of **3**
Figure S4: CID mass spectrum of **4**
Figure S5: CID mass spectrum of **5**
Figure S6: CID mass spectrum of **6**
Figure S7: CID mass spectrum of **7**
Figure S8: CID mass spectrum of **8**
Figure S9: CID mass spectrum of **9**
Figure S10: CID mass spectrum of **10**
Figure S11: CID mass spectrum of **11**
Figure S12: CID mass spectrum of **12**
Figure S13: CID mass spectrum of **13**
Figure S14: CID mass spectrum of **14**
Figure S15: CID mass spectrum of **15**

## Data Availability

Data available on request from the authors.
