# Supplementary material for: Electrospray ionization–tandem mass spectrometric study of fused nitrogen‐containing ring systems
Source: J Mass Spectrom. 2022 Jun 9;57(6):e4870. doi: 10.1002/jms.4870 (PMC9285442; doi:10.1002/jms.4870)
Supplement: Supplementary file 1 — Figure S1: CID mass spectrum of 1 Figure S2: CID mass spectrum of 2 Figure S3: CID mass spectrum of 3 Figure S4: CID mass spectrum of 4 Figure S5: CID mass spectrum of 5 Figure S6: CID mass spectrum of 6 Figure S7: CID mass spectrum of 7 Figure S8: CID mass spectrum of 8 Figure S9: CID mass spectrum of 9 Figure S10: CID mass spectrum of 10 Figure S11: CID mass spectrum of 11 Figure S12: CID mass spectrum of 12 Figure S13: CID mass spectrum of 13 Figure S14: CID mass spectrum of 14 Figure S15: CID mass spectrum of 15 [file JMS-57-0-s001.docx]

**Supporting information**

**Electrospray ionization-tandem mass spectrometric study of fused nitrogen-containing ring systems**

Gábor Krajsovszky^1^, Borbála Dalmadiné Kiss^2^, Krisztina Ludányi^2^, István M. Mándity^1,3^, Dóra Bogdán^1,3^

*^1^ Department of Organic Chemistry, Semmelweis University, Hőgyes E. u. 7,*

*H-1092 Budapest, Hungary*

*^2^ Department of Pharmaceutics, Semmelweis University, Hőgyes E. u. 7,*

*H-1092 Budapest, Hungary*

*^3^ Artificial Transporters Research Group, Research Centre for Natural Sciences, Institute of Materials and Environmental Chemistry, Magyar tudósok körútja 2, H-1117 Budapest, Hungary*

Fig. S1: CID mass spectrum of **1**


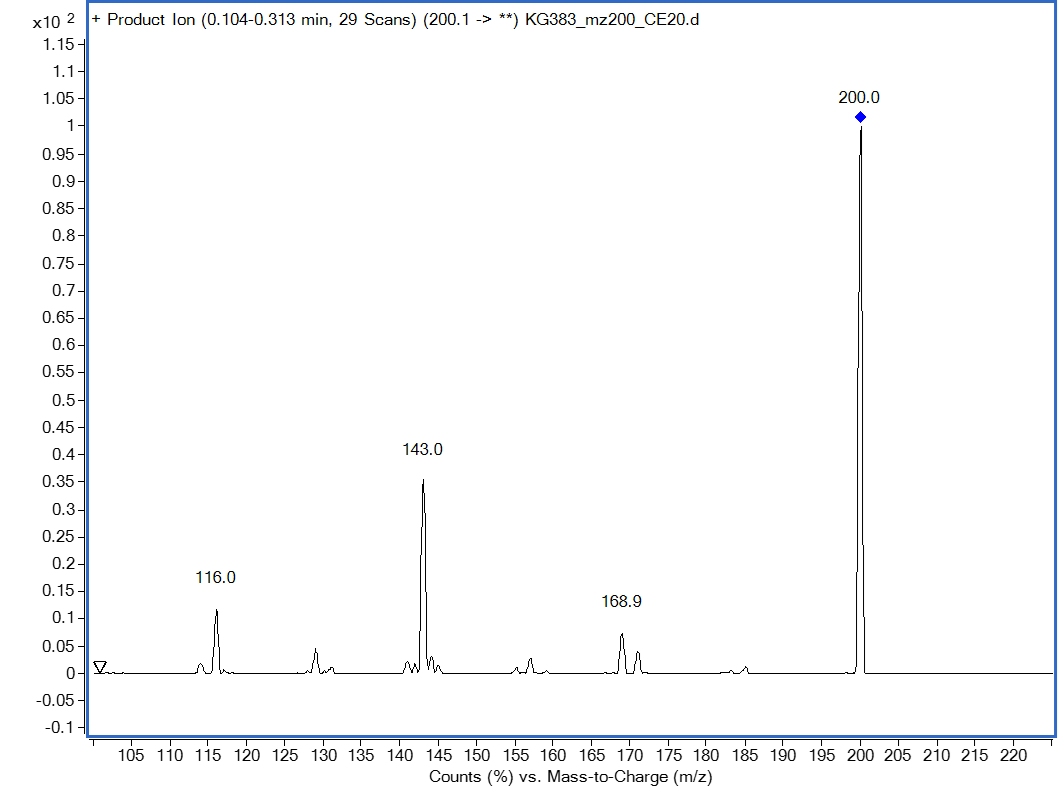


| *m/z* | Relative abundance (%) |
| --- | --- |
| 200.0 | 100.0 |
| 168.9 | 7.2 |
| 143.0 | 35.7 |
| 116.0 | 10.7 |

Fig. S2: CID mass spectrum of **2**


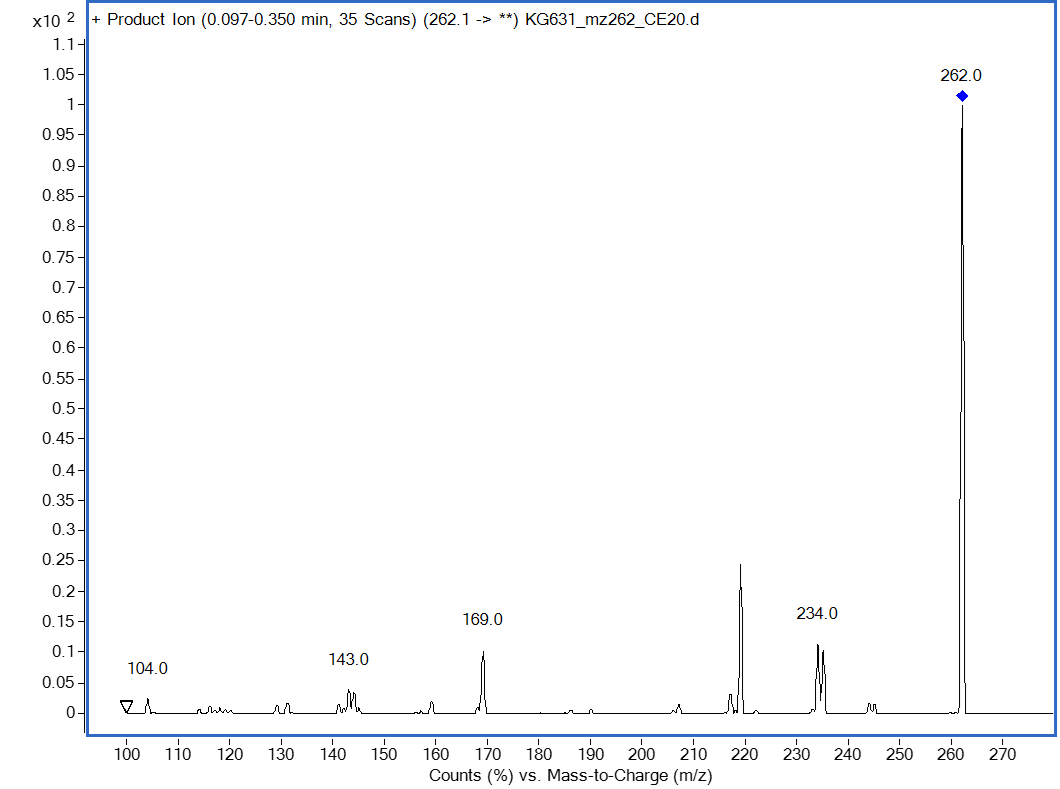


| *m/z* | Relative abundance (%) |
| --- | --- |
| 262.0 | 100.0 |
| 234.0 | 12.2 |
| 169.0 | 11.1 |
| 143.0 | 4.2 |
| 104.0 | 3.7 |

Fig. S3: CID mass spectrum of **3**

**
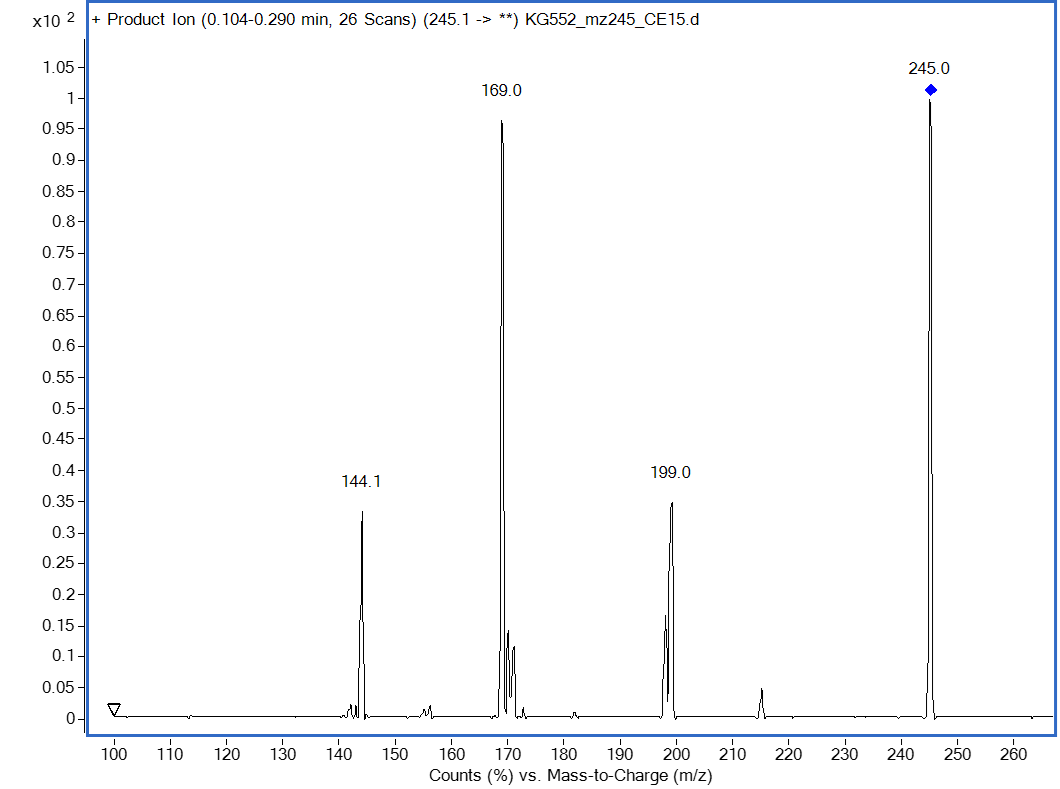
**

| *m/z* | Relative abundance (%) |
| --- | --- |
| 245.0 | 100.0 |
| 199.0 | 36.4 |
| 169.0 | 92.9 |
| 144.1 | 34.1 |

Fig. S4: CID mass spectrum of **4**


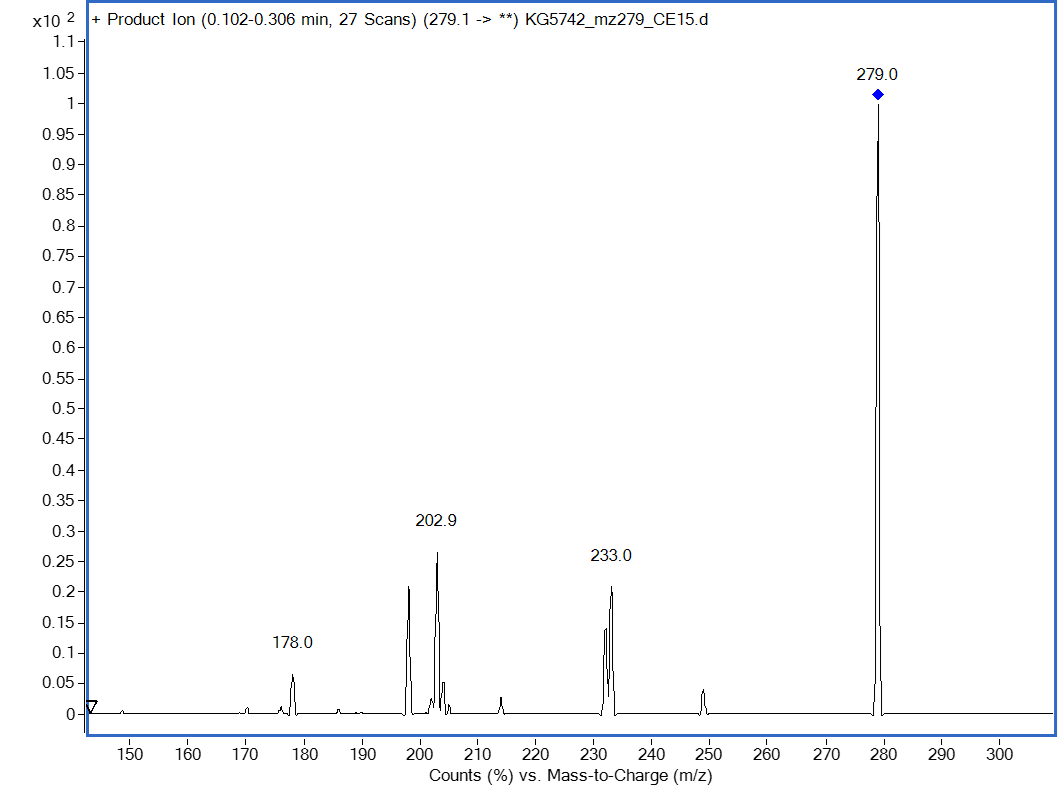


| *m/z* | Relative abundance (%) |
| --- | --- |
| 279.0 | 100.0 |
| 233.0 | 21.2 |
| 202.9 | 27.4 |
| 178.0 | 7.7 |

Fig. S5: CID mass spectrum of **5**


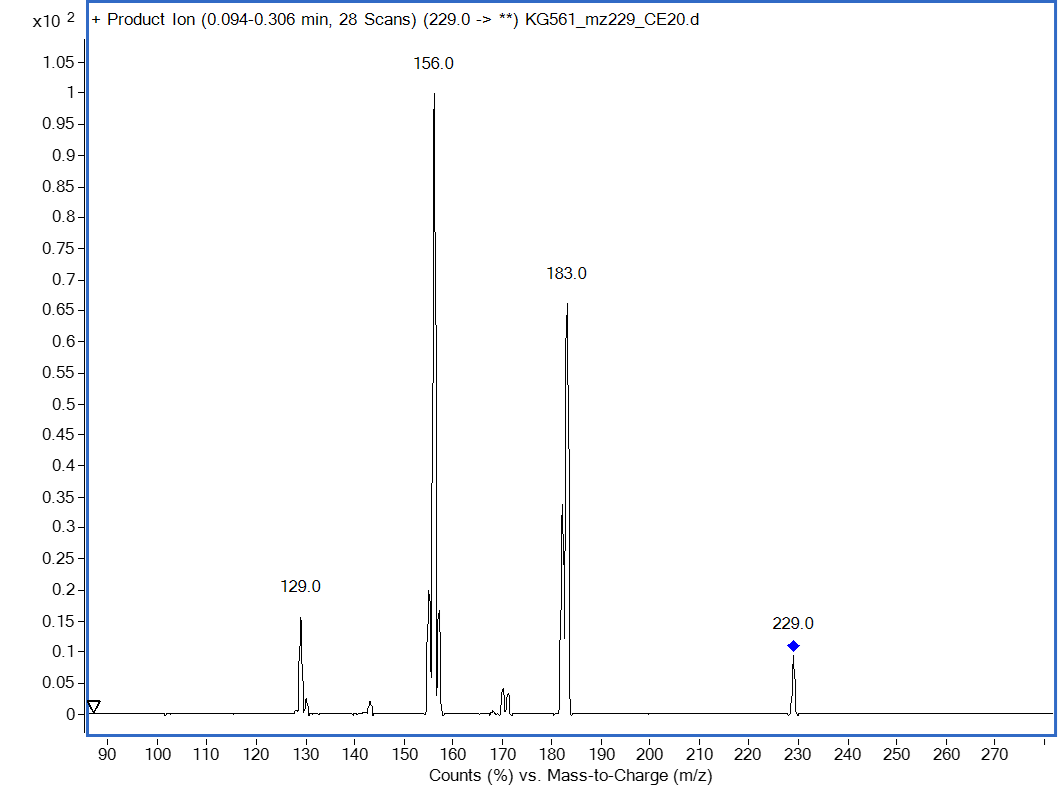


| *m/z* | Relative abundance (%) |
| --- | --- |
| 229.0 | 10.1 |
| 183.0 | 65.6 |
| 156.0 | 100.0 |
| 129.0 | 15.2 |

Fig. S6: CID mass spectrum of **6**

**
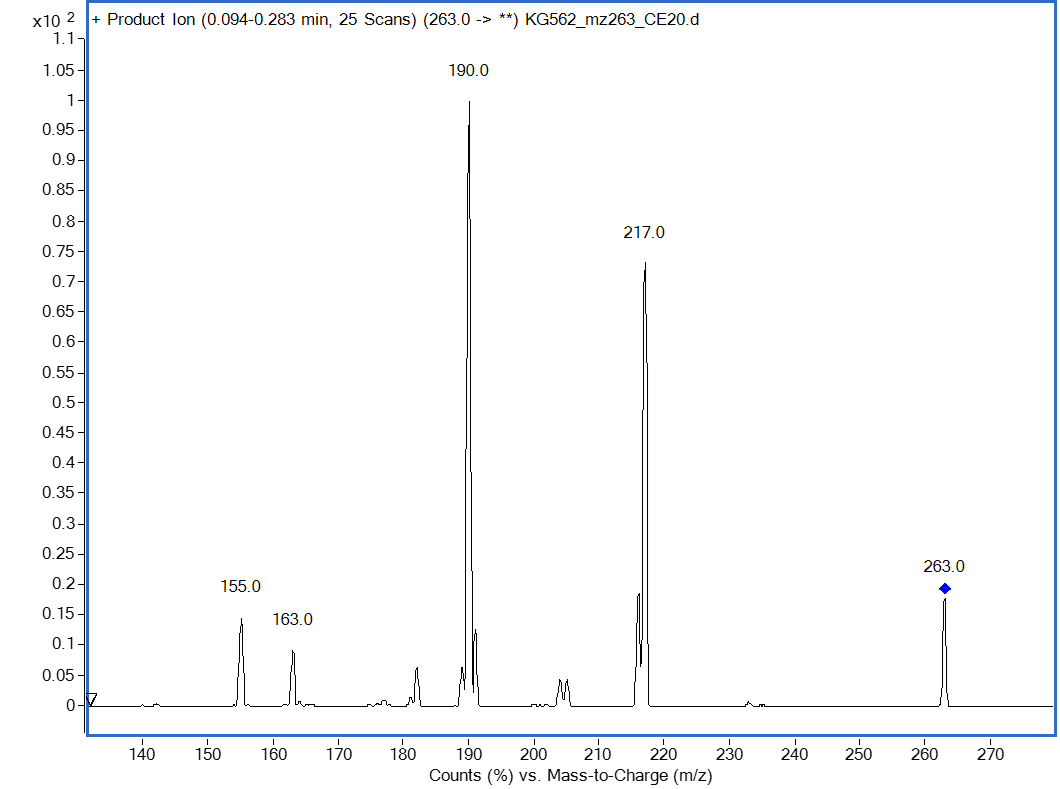
**

| *m/z* | Relative abundance (%) |
| --- | --- |
| 263.0 | 18.5 |
| 217.0 | 74.1 |
| 190.0 | 100.0 |
| 163.0 | 9.3 |
| 155.0 | 14.8 |

Fig. S7: CID mass spectrum of **7**

**
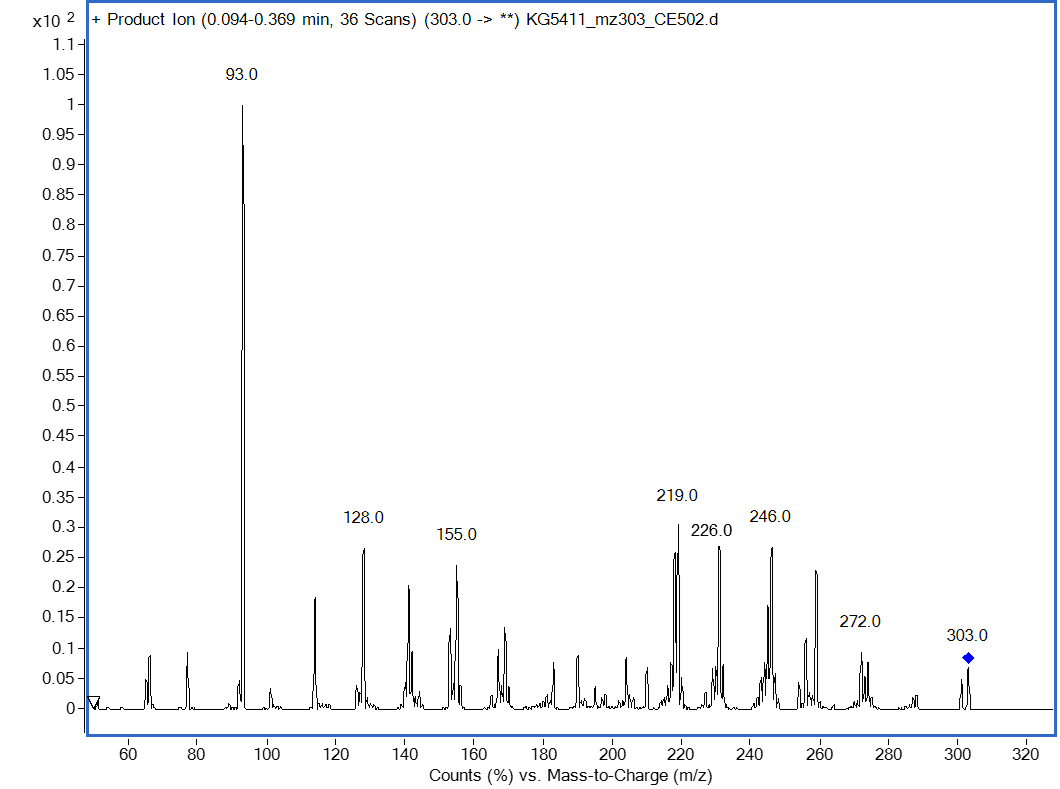
**

| *m/z* | Relative abundance (%) |
| --- | --- |
| 303.0 | 9.3 |
| 272.0 | 8.8 |
| 246.0 | 25.5 |
| 226.0 | 25.4 |
| 219.0 | 29.4 |
| 155.0 | 24.2 |
| 128.0 | 25.8 |
| 93.0 | 100.0 |

Fig. S8: CID mass spectrum of **8**

**
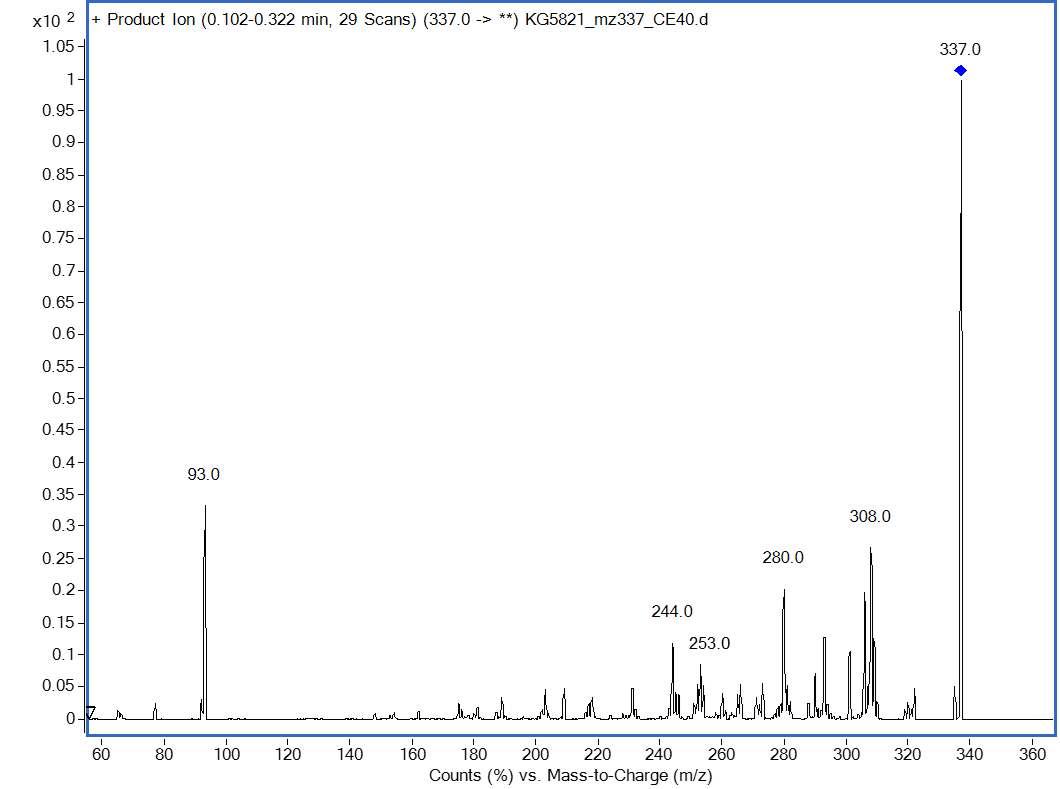
**

| *m/z* | Relative abundance (%) |
| --- | --- |
| 337.0 | 100.0 |
| 308.0 | 28.2 |
| 280.0 | 20.9 |
| 253.0 | 8.1 |
| 244.0 | 12.3 |
| 93.0 | 34.9 |

Fig. S9: CID mass spectrum of **9**


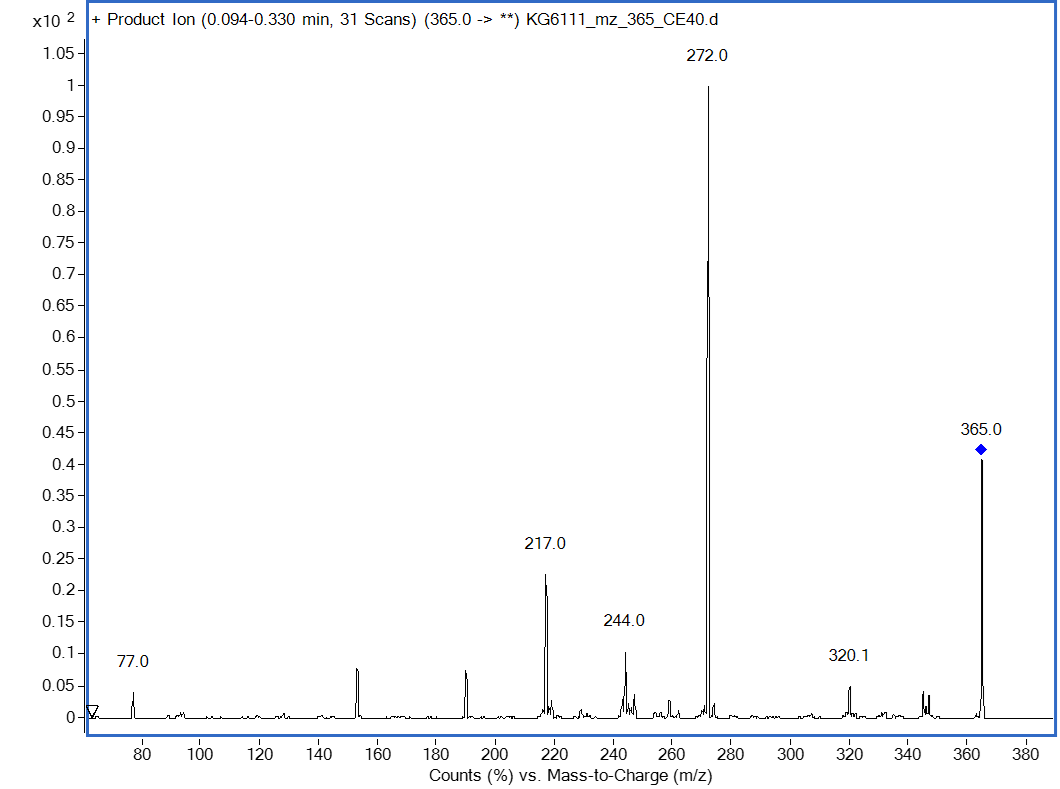


| *m/z* | Relative abundance (%) |
| --- | --- |
| 365.0 | 42.2 |
| 320.1 | 4.8 |
| 272.0 | 100.0 |
| 244.0 | 10.9 |
| 217.0 | 23.4 |
| 77.0 | 4.1 |

Fig. S10: CID mass spectrum of **10**


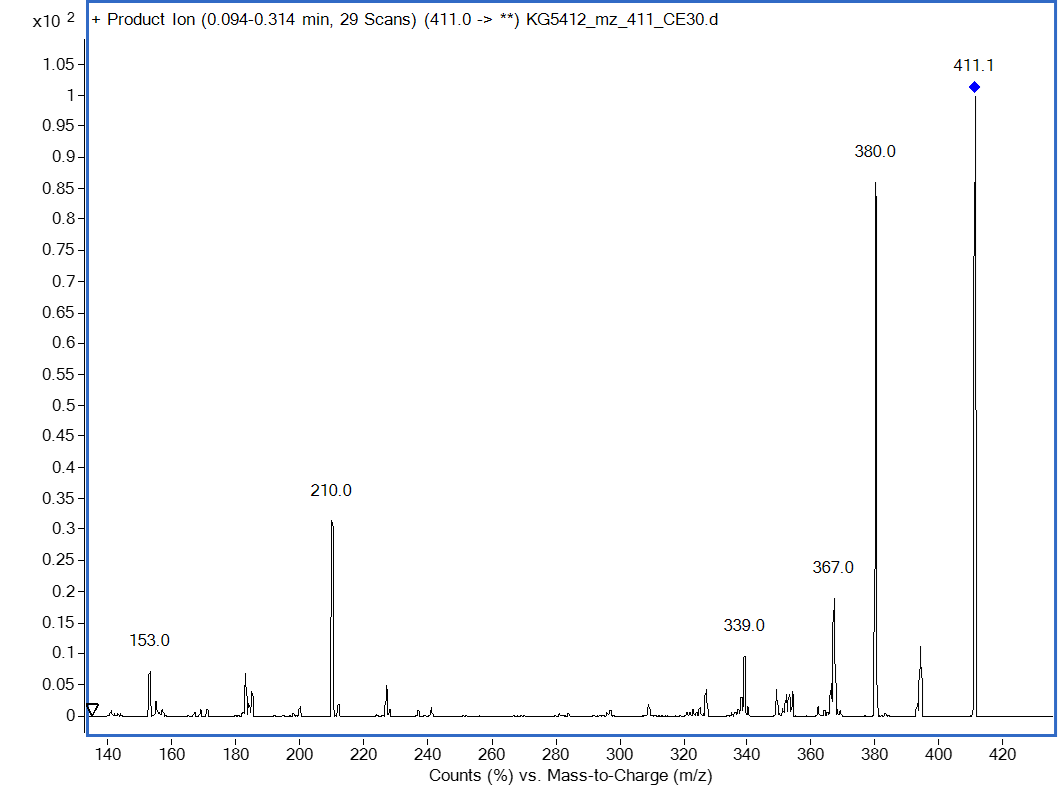


| *m/z* | Relative abundance (%) |
| --- | --- |
| 411.1 | 100.0 |
| 380.0 | 88.1 |
| 367.0 | 19.2 |
| 339.0 | 11.9 |
| 210.0 | 34.0 |
| 153.0 | 7.8 |

Fig. S11: CID mass spectrum of **11**


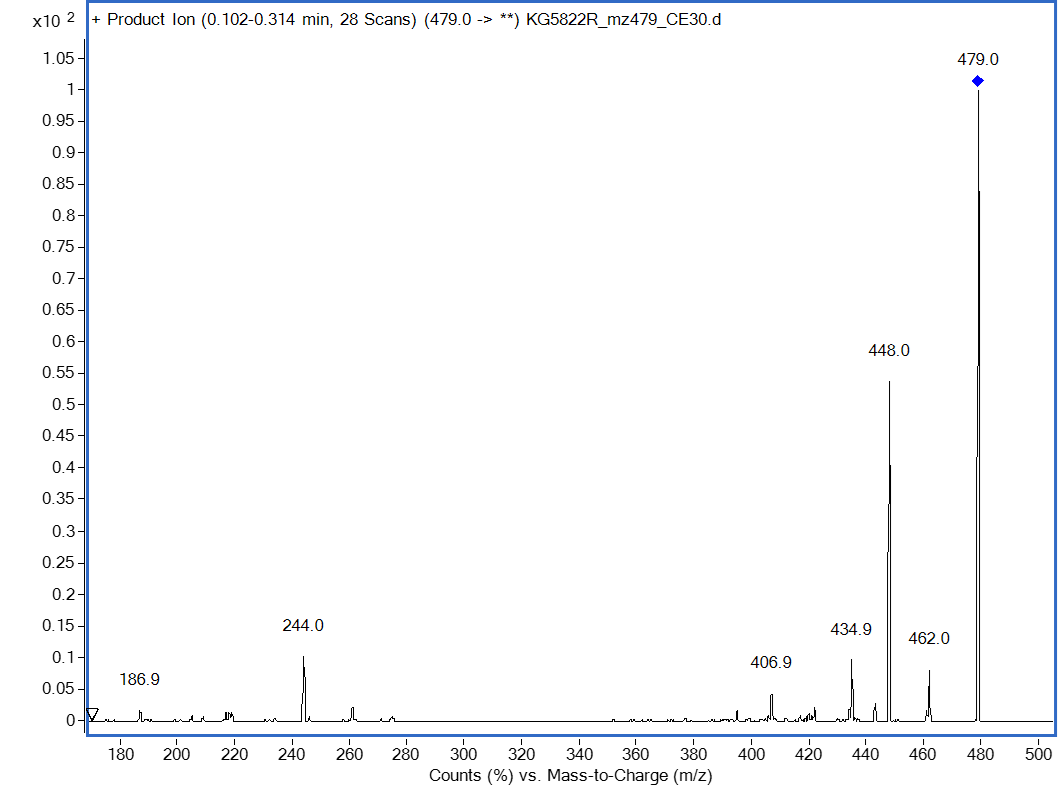


| *m/z* | Relative abundance (%) |
| --- | --- |
| 479.0 | 100.0 |
| 462.0 | 8.2 |
| 448.0 | 55.1 |
| 434.9 | 12.4 |
| 406.9 | 4.5 |
| 244.0 | 10.1 |
| 186.9 | 2.3 |

Fig. S12: CID mass spectrum of **12**

**
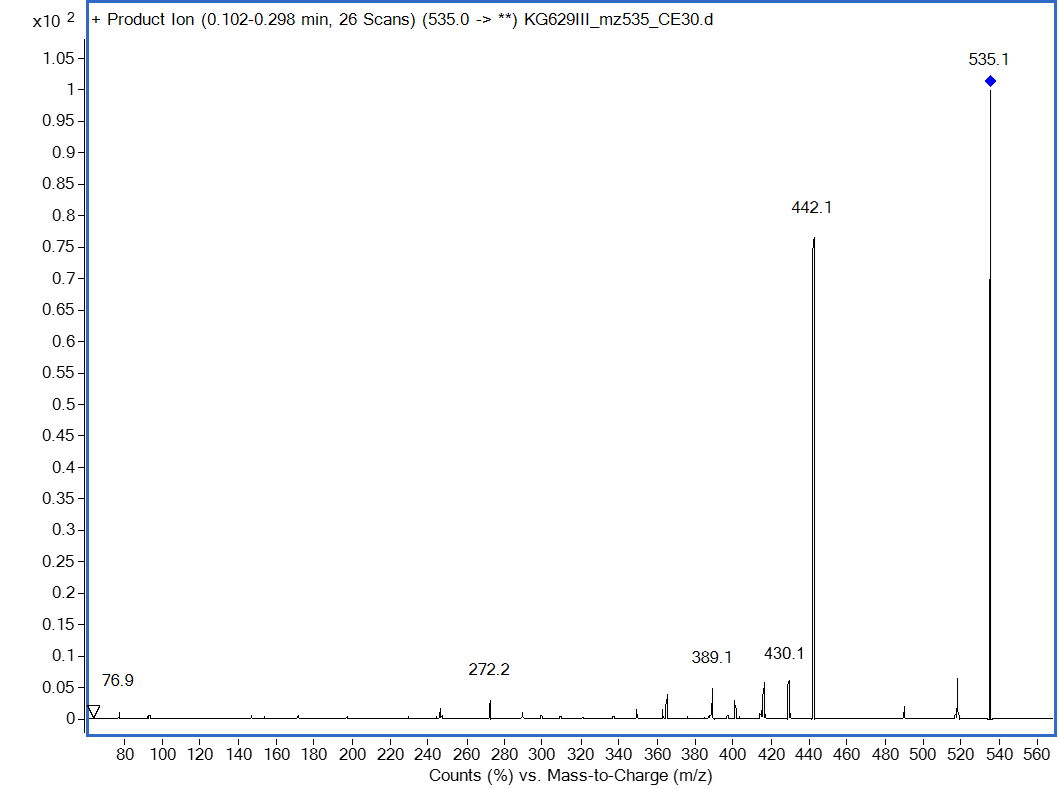
**

| *m/z* | Relative abundance (%) |
| --- | --- |
| 535.1 | 100.0 |
| 442.1 | 78.2 |
| 430.1 | 5.6 |
| 389.1 | 4.8 |
| 272.2 | 2.8 |
| 76.9 | 1.9 |

Fig. S13: CID mass spectrum of **13**

**
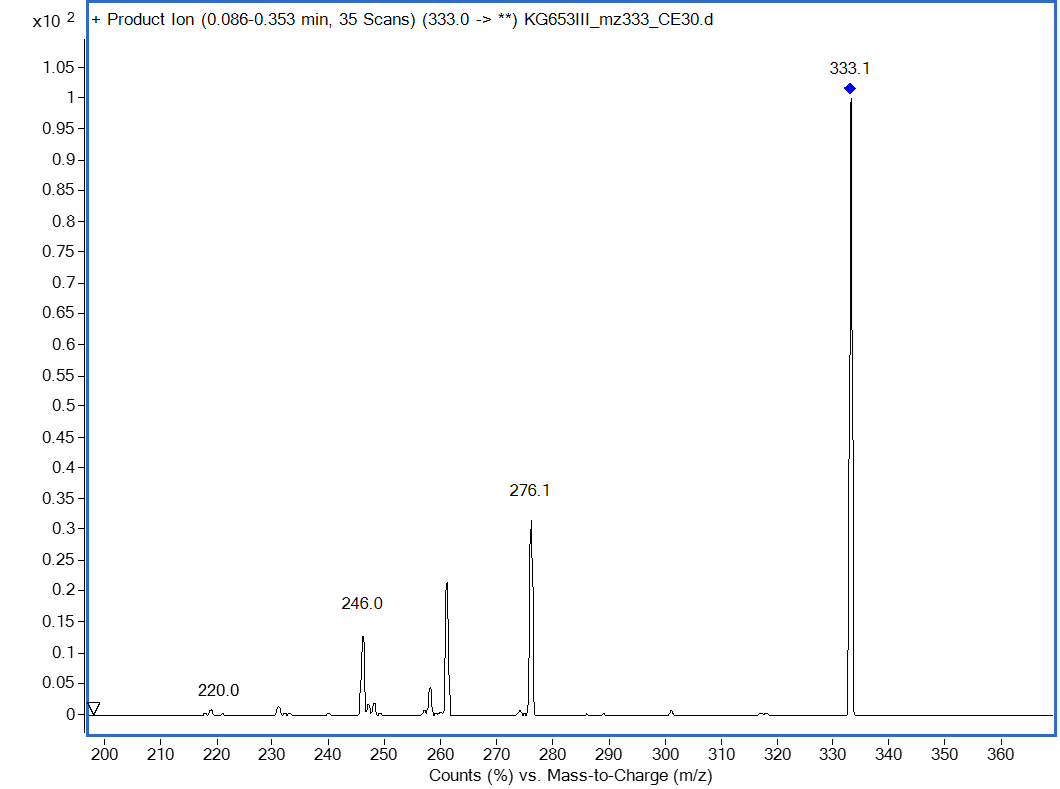
**

| *m/z* | Relative abundance (%) |
| --- | --- |
| 333.1 | 100.0 |
| 276.1 | 33.6 |
| 246.0 | 14.8 |
| 220.0 | 1.6 |

Fig. S14: CID mass spectrum of **14**

**
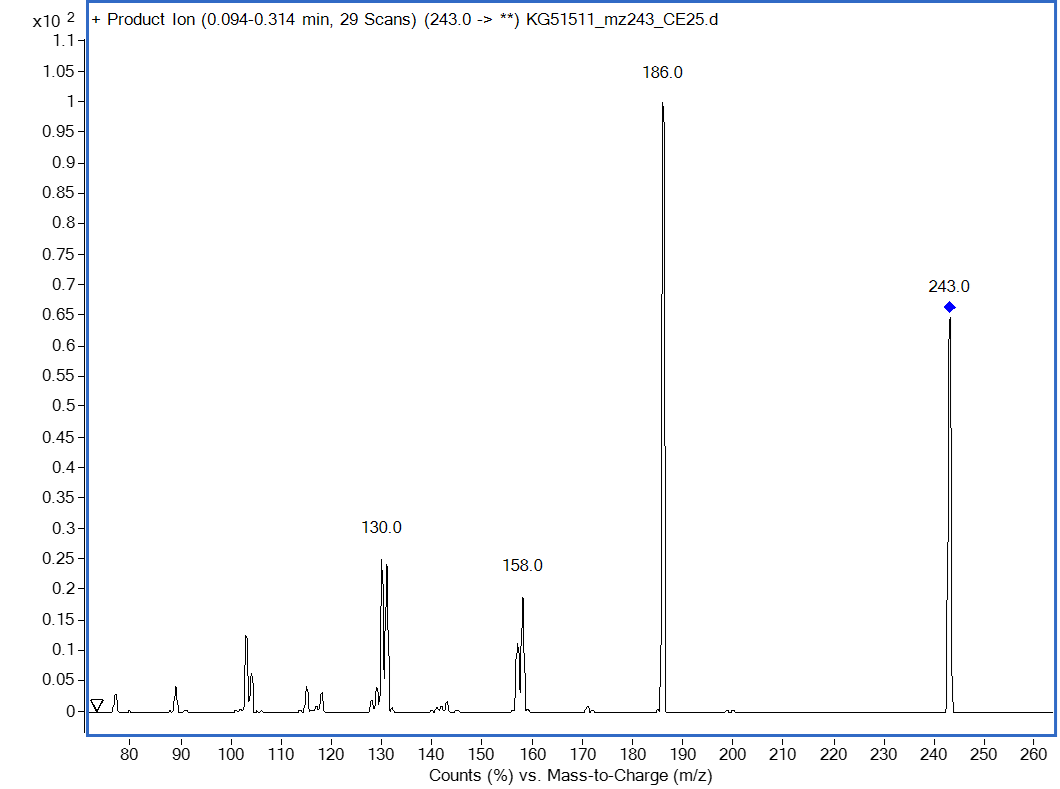
**

| *m/z* | Relative abundance (%) |
| --- | --- |
| 243.0 | 66.7 |
| 186.0 | 100.0 |
| 158.0 | 21.8 |
| 130.0 | 25.9 |

Fig. S15: CID mass spectrum of **15**


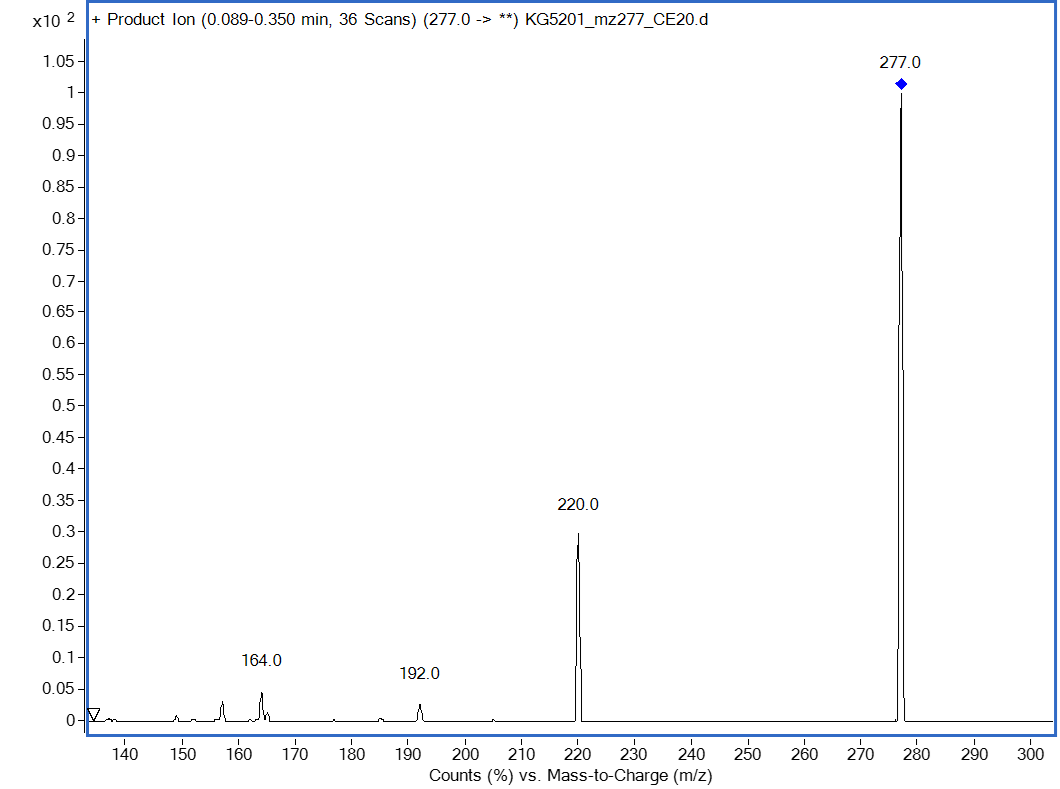


| *m/z* | Relative abundance (%) |
| --- | --- |
| 277.0 | 100.0 |
| 220.0 | 30.6 |
| 192.0 | 4.7 |
| 164.0 | 5.2 |
